# Bacterial metabolic signatures in MASLD predicted through gene-centric studies in stool metagenomes

**DOI:** 10.1186/s12866-025-04549-5

**Published:** 2025-12-18

**Authors:** Juan Manuel Medina-Méndez, Paula Iruzubieta, Raúl Fernández-López, Javier Crespo, Fernando de la Cruz

**Affiliations:** 1 Instituto de Biomedicina y Biotecnología de Cantabria (IBBTEC), Spanish National Research Council (CSIC) - University of Cantabria, Santander, Spain; 2 Gastroenterology and Hepatology Department, Marqués de Valdecilla University Hospital. Clinical and Translational Research in Digestive Diseases, Instituto de Investigación Valdecilla (IDIVAL), Santander, Spain

**Keywords:** MASLD, Gut microbiome, Metagenomics, Metabolic genes, Metabolites, Functional profiling, Plasmids, Accessory genome

## Abstract

**Background:**

Metabolic dysfunction-associated steatotic liver disease (MASLD) is a multifactorial condition in which the gut microbiome (GM) plays a central role. However, taxonomic associations derived from 16S ribosomal RNA (rRNA) gene studies have yielded inconsistent results, likely due to limited resolution and functional redundancy across taxa. We aimed to identify robust, functionally relevant microbial markers of MASLD using metagenomics and gene-centric profiling.

**Methods:**

We analyzed 554 fecal metagenomes from three independent cohorts. Sequencing reads were quality-controlled and taxonomically profiled with multi-marker gene resolution. We quantified the abundance of over 50 target gene families involved in butyrate, methane, trimethylamine (TMA) and short-chain alcohol (SCAs, i.e., ethanol and propanol) metabolism. Their presence was also determined across complete GM genomes and plasmids.

**Results:**

Genes involved in butyrate and methane production tended to show lower abundance in MASLD, particularly in cirrhosis, while TMA- and SCA-producing genes were frequently enriched. These functional shifts were accompanied by the depletion of *Agathobacter rectalis*. Many of the altered genes were highly accessory and encoded on plasmids, suggesting genome-specific functional divergence driven by horizontal gene transfer.

**Conclusion:**

MASLD is characterized by a shift toward alcohol- and TMA-producing metabolism, alongside reduced butyrate and methane production -changes driven by accessory and plasmid-borne genes. Gene-centric and mobile genetic element-aware profiling reveals mechanistic microbial contributions to MASLD that remain undetected by taxonomy-based approaches, offering new targets for diagnosis and intervention.

**Supplementary Information:**

The online version contains supplementary material available at 10.1186/s12866-025-04549-5.

## Highlights

• MASLD is linked to consistent, gene-level functional shifts in the gut microbiome.

• Genes involved in butyrate and methane synthesis are depleted in MASLD patients.

• Genes encoding microbial ethanol, propanol and TMA production are enriched.

• Many of these metabolic genes are plasmid-encoded, revealing strain-level divergence.

• Functional changes occur independently of major taxonomic shifts.

**Supplementary Information:**

The online version contains supplementary material available at 10.1186/s12866-025-04549-5.

## Impact and implications

We found that MASLD is marked by distinct geno-metabolic shifts in the GM, including the enrichment of plasmid-encoded genes involved in the production of TMA and endogenous alcohols. These alterations reflect functional contributions to disease that escape taxonomic profiling. By highlighting the role of horizontally-transferred, accessory genes in shaping host-microbiome interactions, our results underscore the importance of gene-centric frameworks in microbiome research. Since horizontal gene transfer drives such functional and accessory gene diversity, similar mechanisms may contribute to other host-microbiome pathologies.

## Background

Metabolic dysfunction-associated steatotic liver disease (MASLD) is the most common chronic liver disease worldwide, affecting approximately 38% of the adult population, with an increasing prevalence largely driven by the global rise in obesity and type 2 diabetes mellitus [1]. MASLD encompasses a spectrum from simple steatosis to metabolic dysfunction-associated steatohepatitis (MASH), which can further progress to fibrosis, cirrhosis and hepatocellular carcinoma [2]. Beyond hepatic complications, MASLD is strongly associated with cardiovascular disease, extrahepatic cancers and chronic kidney disease, highlighting its role as a multisystemic metabolic disorder [3]. The pathogenesis of MASLD is complex and multifactorial, involving genetic susceptibility, environmental exposures, inflammatory and metabolic factors, with a strong association with insulin resistance [4].

Over the past decade, the gut-liver axis (GLA) has emerged as a central player in MASLD progression. Alterations in the gut microbiome (GM) composition and function have been linked to increased intestinal permeability [5], microbial translocation, and hepatic exposure to endotoxins and bioactive microbial metabolites, including lipopolysaccharide [6], short-chain fatty acids (SCFAs) and trimethylamine-N-oxide (TMAO). These microbial-derived products derive from undigested dietary carbohydrates and proteins that are fermented by the GM in the colon [7], and they influence lipid metabolism, insulin signaling, hepatic inflammation and fibrogenesis through both direct and indirect mechanisms [8].

Several human and preclinical studies have reported compositional alterations in the GM of MASLD patients using both metabolomic and taxonomic approaches [9–12]. Increased abundances have been observed in certain bacterial clades, including *Enterobacteriaceae* [12–14], *Bacteroidaceae* [14, 15], *Veillonellaceae* [12] and the genera *Streptococcus* [16], *Fusobacterium*, *Bilophila*, *Dorea* and *Peptoniphilus* [17, 18]. Conversely, other clades have been reported to be reduced, such as *Rickenellaceae* and *Ruminococcaceae*, as well as the genera *Clostridium* [13], *Pseudomonas* [16], *Coprococcus*, *Akkermansia* and the species *Faecalibacterium prausnitzii* [14, 19], *Eubacterium rectale* [14, 19] or *Dorea longicatena* [19]). However, taxonomic associations across studies remain inconsistent [17, 20].

In parallel, metabolomic analysis demonstrated that SCFAs, including acetate and butyrate, were significantly lower in MASLD patients across all stages, with more pronounced reductions observed in moderate-to-severe cases [9]. Nevertheless, metabolite levels can be influenced by host metabolism, age, diet and environmental factors [21], while taxonomic profiling based on 16S ribosomal (rRNA) gene often lacks resolution at or below species-level. As a consequence, it may fail to capture functional capabilities such as key metabolic pathways, especially those related to SCFA biosynthesis, which are sometimes widely distributed across diverse taxa and, thus, not taxonomically restricted. In fact, bacterial genomic plasticity allows key metabolic genes to be encoded in their accessory genome, including mobile genetic elements (MGEs), further decoupling taxonomic identity and metabolic function [22].

Given these limitations, there is increasing recognition of the value of function-centric strategies, including metagenomics and gene-centric profiling [23], to better elucidate the microbial mechanisms underpinning MASLD. These methods allow for direct quantification of microbial gene families involved in key metabolic pathways and enable the detection of accessory and plasmid-borne genes that may be decoupled from microbial taxonomy.

In this study, we used high-resolution metagenomic data from three independent cohorts to investigate the microbial genes encoding enzymes involved in the final steps of pathways leading to the production of butyrate, short-chain alcohols (SCAs) ethanol and propanol, methane, trimethylamine (TMA) and TMAO, all key metabolic routes implicated in MASLD pathophysiology. We also explored taxonomic signatures to assess bacterial diversity and abundance. Finally, we examined the distribution of these genes within the human GM genomes and plasmids. By focusing on functionally relevant genes and MGEs, we aim to move beyond correlative taxonomic observations to provide mechanistic insights into the functional reprogramming of the GM in MASLD. This gene-centric framework offers new perspectives of the microbial contributions to MASLD progression and suggests new targets for diagnosis and intervention.

## Methods

### Description of MASLD patient cohorts

Cohort 1 includes 83 European MASLD patients with obesity and early-stage fibrosis (F0-F2), aged 20–64 years. Patients were drawn from two previously published studies: 73 individuals from Hoyles et al. [24] and 10 from Mardinoglu et al. [25]. MASLD diagnoses were confirmed by liver biopsy performed during bariatric surgery. None of the subjects had alcoholic liver disease, viral hepatitis or diabetes mellitus. The control group includes 117 healthy individuals from Poyet et al. [26]. Cohort 2 comprises 86 American MASLD patients from Loomba et al. [14], with 72 exhibiting early-stage fibrosis and 14 with advanced fibrosis (F3-F4), diagnosed by liver biopsy and magnetic resonance imaging. No participants had liver comorbidities or diabetes. For analysis, patients with early-stage fibrosis were treated as a “control” group. Cohort 3 includes 230 Han Chinese individuals from Qin et al. [27]: 125 diagnosed with MASLD-related cirrhosis and 105 without liver injury. Patients with viral hepatitis or other hepatic disorders were excluded. In total, five studies were included (PRJNA544527, PRJEB14215, PRJNA420817, PRJNA373901, and PRJEB6337), which were stratified into three cohorts (Table 1).

**Table 1:** MASLD patient cohorts analyzed in this study. Each cohort (column 1) was divided into two comparison groups: control vs. disease (column 2). Column 3 shows the number of metagenomic stool samples analyzed from the MASLD patients. Original studies are listed in column 4, with metagenomic data and clinical metadata retrieved from the SRA accession numbers in column 5

Cohort	Phenotypic group	*N* samples	Reference	SRA accession number
1	Healthy control	117	[26]	PRJNA544527
	MASLD, early-stage fibrosis (F0-F2)	73	[24]	PRJEB14215
		48	[25]	PRJNA420817
2	MASLD, early-stage fibrosis (F0-F2)	72	[14]	PRJNA373901
	MASLD, advanced fibrosis (F3-F4)	14		
3	Healthy control	105	[27]	PRJEB6337
	MASLD-related cirrhosis	125		

### Retrieval of metagenomic sequencing data

A total of 554 metagenomic sequencing libraries generated from fecal samples of MASLD patients and healthy subjects were retrieved from the Sequence Read Archive (SRA) using SRA Toolkit (version 3.2) [28] (Supplementary Fig. 1). For Cohort 1, the 83 MASLD patients included 73 from Hoyles et al. and 10 from Mardinoglu et al., which contributed 48 stool samples, as indicated in Table 1. Public studies were selected based on the availability of metagenome shotgun sequencing data from fecal samples together with sufficient medical metadata to verify MASLD diagnosis.

Medical metadata referred primarily to diagnostic status (MASLD vs. healthy control) and fibrosis stage (F0-F4), which were obtained either from the BioSample annotations in the SRA. Samples were classified into two phenotypic groups per cohort, and those with undefined MASLD status or missing diagnostic information were excluded. Information on sample collection, DNA extraction and sequencing procedures is provided in the original studies. Data retrieval was performed with the SRA Toolkit using accession lists containing the SRA run identifiers for each study. The resulting FASTQ files served as input for downstream processing. The following commands were used:

- prefetch --option-file accessions.txt --output-directory./SRA
- fastq-dump --split-files --outdir ./FASTQ./SRA/PRJXXXXXXX.sra

### Quality control of metagenomic reads

Sequencing reads were trimmed to remove Illumina adapters and low-quality regions (< Q25) using BBDuk from the BBTools suite (version 37.62) [29]. Minimum read-length cutoffs were applied on a per-dataset basis, adjusted according to the quality profile of each dataset to ensure reliable downstream analysis. Read quality was assessed with FastQC (version 0.12.1) [30].

### Taxonomic signatures associated with MASLD

Taxonomic profiling was performed with MetaPhlAn (version 4.1.1), which uses a wide-range of clade-specific marker genes to estimate GM abundances [31]. We used the *mpa_vJan25_CHOCOPhlAnSGB_202503* database (downloaded on September 29, 2025) and added the *--ignore_eukaryotes* flag. Post-processing involved removing clades predicted as “unclassified” and clades with zero mean abundance across all samples in any cohort, while retaining clades that were present exclusively in one of the comparison groups to preserve potential very clear taxonomic markers. Genera with >0.5% and species with >0.2% average abundance were retained.

### Isolation of target gene families

To investigate pathways producing butyrate, ethanol, propanol, TMA, methane and their precursors, we constructed the corresponding gene families from the Unified Human Gastrointestinal Genome (UHGG, version 2.0.2) [32]. Candidate genes were defined from UHGG pangenomes based on gene annotations, using custom scripts that extracted headers matching gene name patterns and allowed exclusion of spurious hits via extended negative matching. Sequences were cross-checked against species-representative proteomes to confirm consistency with the expected enzyme annotation.

Recovered sequences were filtered by length and aligned with MAFFT (version 7.271) [33] using options *“--localpair”* and *“--maxiterate 1000”*. To remove spurious or fragmented sequences, alignments were processed with a gap filter that excluded sequences with ≥ 30% gaps. Each seed alignment was concatenated with an experimentally validated reference enzyme obtained from MetaCyc [34], ensuring that family delimitation was anchored to an experimentally validated sequence.

Maximum-likelihood trees were reconstructed with IQ-TREE (version 2.0.3) [35], and family membership was refined according to clustering around the reference sequences. Profile Hidden Markov Models (pHMMs) were then constructed using the *hmmbuild* and *hmpress* modules from HMMER (version 3.4) [36]. Functional annotation was cross-checked through MetaCyc [34] and KEGG [37], considering gene names, enzymatic reactions, KEGG Orthology (KO) assignments and enzyme commission (EC) numbers.

In parallel, a set of five universal single-copy genes (USCGs) were retrieved using the same procedure, to serve as internal controls: *argS* (arginyl-tRNA synthetase), *dnaA* (chromosomal replication initiator protein DnaA), *rpoA*, *rpoB* and *rpoC* (alpha, beta and gamma subunits of the DNA-directed RNA polymerase, respectively). The scripts implementing this pipeline is available at https://github.com/JuanmaMedina/MASLD_FG_paper for full reproducibility.

### Abundance analysis of target genes in metagenomes

All metagenomic reads used for abundance analysis had passed quality control, with minimum read-length cutoffs applied individually per dataset based on its quality profile (see Section C). This ensured that only high-quality reads were retained for downstream mapping. Gene abundances were then estimated by aligning quality-controlled reads against the isolated gene families using DIAMOND (version 2.0.14) [38]. Alignments were filtered to retain the best match per query read and gene sequence using *“--max-hsps 1”* and an E-value < 1E-10, following validated parameters for fecal metagenomes [39]. The resulting alignments were parsed to count the number of reads mapping to each gene family, which provided absolute abundance estimates.

Abundance values were then normalized using a metric based on the number of reads per kilobase per family size per million reads (RPKSM), which is a modified version of the RPKM metric [40] that accounts for gene length, family size and library size. Specifically, S represents the number of individual genes comprising each gene family (e.g., subunits or paralogs), enabling abundance comparisons between families of different sizes. The full workflow, including the DIAMOND-based mapping and quantification script, is available at https://github.com/JuanmaMedina/MASLD_FG_paper, ensuring reproducibility.

### Whole metagenomic analysis

We applied a whole-metagenome analysis workflow to validate our results at single-gene level. To this end, both HUMAnN (version 3.9 [41]), and SqueezeMeta (version 1.6.0 [42]), were used for functional profiling of metagenomic samples from the three cohorts. Due to computational limitations, approximately 24–30 samples per cohort were randomly selected, ensuring balanced representation of all phenotypic groups. The SqueezeMeta pipeline performed co-assembly, open-reading frame (ORF) prediction, and KO annotation to quantify gene family abundances per sample. The resulting data were further processed with SQMtools (version 1.6.0 [43]), to identify differentially abundant KOs between phenotypic groups within each cohort using DESeq2 (version 1.34.0 [44]), applying a log-fold change >2 and a p-adjusted value < 0.05 as thresholds. In parallel, an exploratory analysis with HUMAnN was conducted to obtain species-resolved functional profiles (results not shown).

### Presence of candidate genes in human GM genomes and plasmids

We assessed the presence of target metabolic genes across UHGG genomes and RefSeq232 plasmids [45]. The plasmid dataset was curated to exclude partial sequences, unassignable hosts, and PacBio internal controls, following previous protocols [22]. Only UHGG genomes with >95% completeness and all five USCGs were retained (*n* = 31,227). ORFs were predicted using Prodigal (version 2.6.3) [46] and screened against pHMMs using HMMER´s *hmmscan* [36]. Hits were retained if they had >90% coverage of the pHMM, E-value < 0.01, and independent E-value < 0.01, minimizing false positives due to similar domains.

Genes were classified as core if present in > 80% of congeneric or conspecific genomes, accessory if in 20–80%, and highly accessory if in < 20%. Only abundant GM clades with > 100 complete genomes were considered. We also evaluated gene presence in annotated plasmids from RefSeq232 (*n* = 110,017 plasmids, > 8,7 million ORFs), applying the same filters and *hmmscan* parameters. Importantly, the taxonomic annotation used in this analysis corresponds to the scheme provided by the UHGG collection, which splits polyphyletic genera such as *Ruminococcus* and *Clostridium* into monophyletic clades (e.g., *Ruminococcus_A*,* Ruminococcus_B*,* Clostridium_Q*). All details on the nomenclature and classification can be found at: https://ftp.ebi.ac.uk/pub/databases/metagenomics/mgnify_genomes/human-gut/v2.0.2/README_v2.0.2.txt

### Statistical analysis

Data analysis was performed using R (version 4.1.3) and the *tidyverse* package (version 1.3.2) [47]. Figures were generated with the R packages *ggplot2* (version 3.4.2) [48], *ggpubr* [49] (version 0.6.0) and *pheatmap* [50] (version 1.0.12). Mann-Whitney tests with a Benjamini-Hochberg adjustment for multiple testing were applied to assess differences in taxonomic and gene abundances between phenotypic groups in each cohort. Statistical analysis and graphical representation on the plots were conducted using the R package *rstatix* (version 0.7.2) [51].

## Results

### Consistent shifts in GM composition in MASLD

**Fig. 1:**
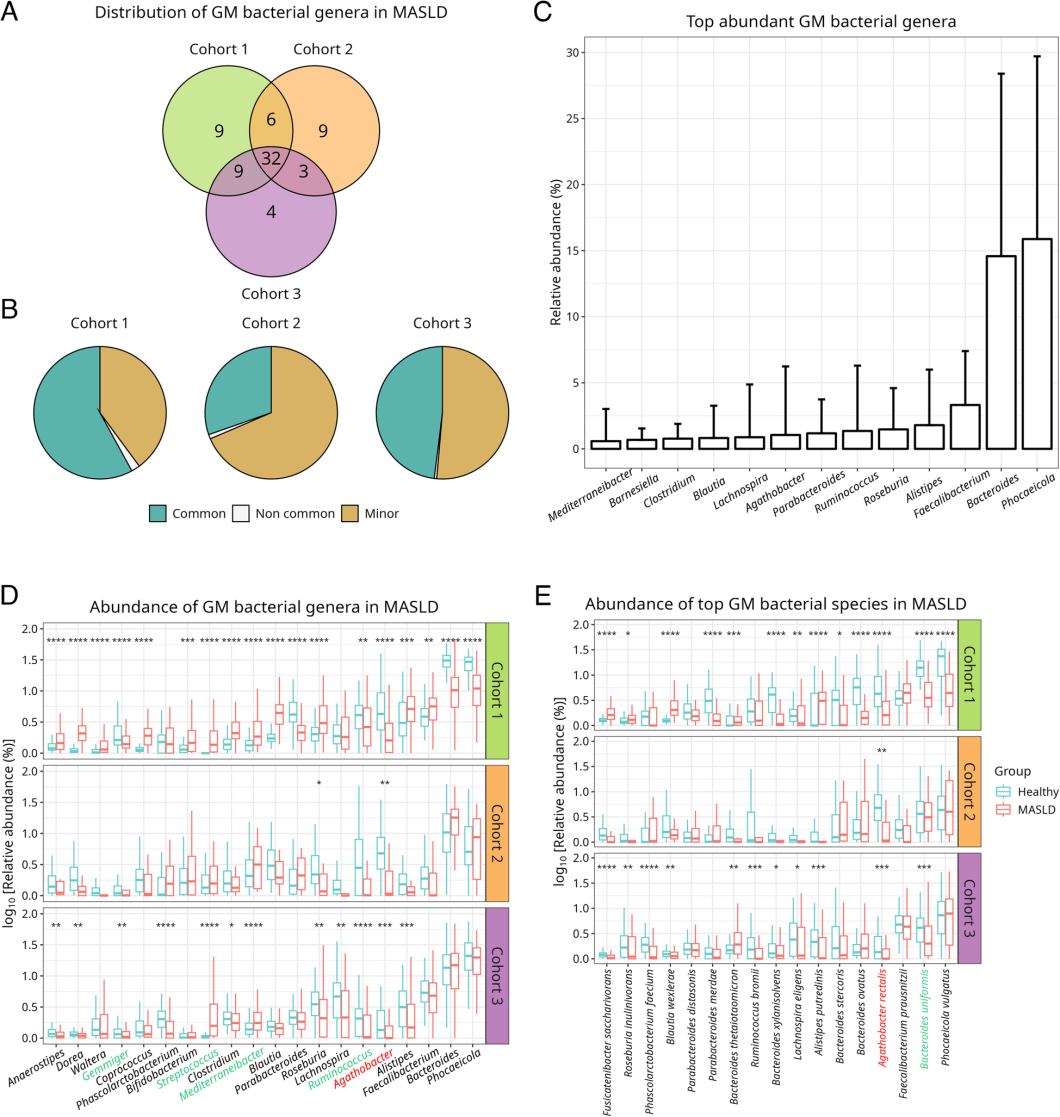
Taxonomic profiling of the GM in MASLD.** A** Number of bacterial genera shared across the three study cohorts. **B** Average relative abundance of genera predicted by MetaPhlAn. Blue sections of the pie charts represent shared genera (*n* = 32), whereas the grey and green sections represent, respectively, cohort-specific and minor genera (i.e., with an average relative abundance of 0 in at least one cohort). **C** Most abundant GM genera (average absolute abundance > 0.5%) (**D-E**) Relative abundance of bacterial genera (**D**) and species (**E**) in fecal metagenomes, shown on a logarithmic scale. From the 32 shared genera shown in A and B, only the 20 most abundant are displayed in D for clarity. Blue and red boxplots indicate healthy and MASLD groups, respectively. Each facet represents one cohort. Differences in abundance were evaluated using pairwise Mann-Whitney tests with Benjamini-Hochberg adjustment. Boxes show the interquartile range, whiskers extend to extend to 1.5 × interquartile, and horizontal bars the medians. Clades in green across the x-axis show consistent group-level trends over cohorts. *Agathobacter rectalis*, in red, is the only species with a statistically significant and consistent depletion in MASLD across all cohorts. (*) *p* < 0.05, (**) *p* < 0.01, (***) *p* < 0.001, (****) *p* < 0.0001

To identify bacterial genera systematically altered in MASLD, we profiled the GM taxonomic composition across three independent subject cohorts (Table 1) at both genus and species level using MetaPhlAn [31], as detailed in Methods. Across cohorts, 32 bacterial genera were commonly detected (Fig. 1A), representing 58%, 30% and 48% of the total GM in Cohorts 1, 2 and 3, respectively (Fig. 1B). Thus, nearly half of the bacterial genera identified in at least one cohort were shared across all three, representing between one-third and two-thirds of their total GM abundance.

Among these shared genera, *Agathobacter*, *Ruminococcus* and *Mediterraneibacter* were not only relatively abundant members of the core GM (Fig. 1C), but also displayed a consistent pattern of differential abundance between MASLD and control groups across all cohorts (Fig. 1D). In fact, five genera -*Gemmiger*, *Streptococcus*, *Mediterraneibacter*, *Ruminococcus* and *Agathobacter*- exhibited a reproducible trend of differential abundance between MASLD and control groups, reaching statistical significance in at least two (Fig. 1D). This threshold was applied to improve robustness and minimize confounding from cohort-specific demographic or environmental variables [21]. Cohort 2 was exempt from this criterion, as it only included MASLD patients, and applying it might have obscured taxonomically relevant trends. Strikingly, *Agathobacter* was the only genus to show significant depletion across all three cohorts (Fig. 1D). This effect was observed in MASLD patients with early-stage disease (F0-F2, Cohort 1), advanced fibrosis (F3-F4, Cohort 2), and cirrhosis (Cohort 3) compared to healthy controls or earlier disease stages (Cohort 2).

At the species level, only *Agathobacter rectalis* and *Bacteroides uniformis*, two of the most abundant GM bacterial species (Supplementary Fig. 2), showed consistent group differences across all cohorts, with significance in at least two (Fig. 1E). Consistently, *A. rectalis* was significantly depleted in MASLD patients in Cohorts 1 and 3, and in early-stage disease patients in Cohort 2 (Supplementary Fig. 3). These findings position *A. rectalis* as a robust, cross-cohort taxonomic indicator of MASLD severity, with potential functional implications given its established role in butyrate production and maintenance of gut barrier integrity [52].

### Microbial butyrate pathways are repressed in progressive MASLD

To explore functional microbial alterations in MASLD, we analyzed the metagenomic fecal samples from the three cohorts for the abundance of bacterial genes involved in butyrate synthesis, using gene-level quantification as a proxy for functional potential independently of taxonomy. Specifically, we quantified the genes encoding the final enzymes of the pathway, as described in Methods.

**Fig. 2:**
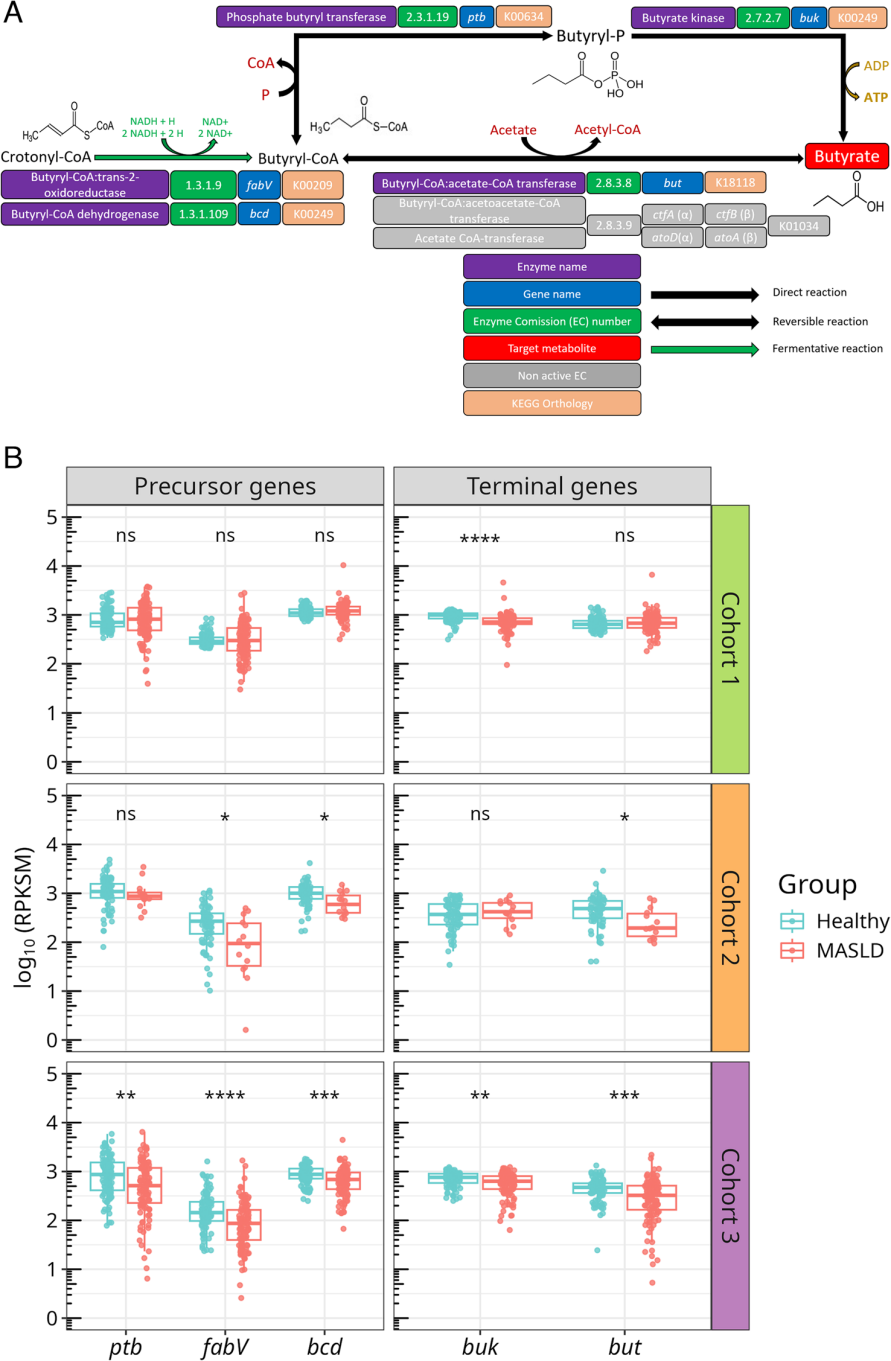
Abundance of butyrate-producing genes in MASLD.** A** Terminal reactions involved in the formation of bacterial butyrate from crotonyl-CoA. Enzymes, EC numbers, coding genes and KOs, as well as reaction types, are indicated according to the legend. **B** Abundance of butyrate-producing genes in fecal metagenomes from the three cohorts, shown on a logarithmic scale. Boxplots show RPKSM values. Boxes represent the interquartile range, whiskers extend to 1.5 × interquartile, and dots indicate individual samples. Differences in abundances were evaluated using pairwise Mann-Whitney tests with Benjamini-Hochberg adjustment. Left and right facets represent abundances of precursor and terminal coding genes, respectively, as described in (A). RPKSM: reads per kilobase per genic size per million reads. (*) *p* < 0.05, (**) *p* < 0.01, (***) *p* < 0.001, (****) *p* < 0.0001, ns: non-significant

Butyrate was selected as a target metabolite due to its well-established role in maintaining colonic epithelial barrier integrity [53], promoting lipid oxidation [54], inducing hepatic gluconeogenesis [55], and preventing insulin resistance [56]. Butyrate is produced by GM through two major terminal pathways (Fig. 2A). The first involves the phosphorylation of butyryl-CoA that is catalyzed by a phosphate butyryl transferase encoded by *ptb*, followed by a dephosphorylation catalyzed via butyrate kinase (*buk*). Alternatively, the CoA-transferase pathway converts butyryl-CoA to butyrate via butyryl-CoA: acetate CoA transferase (*but*), conserving CoA bond energy. Butyryl-CoA itself is produced from crotonyl-CoA through two different enzymes: butyryl-CoA trans-2-oxidorreductase (*fabV*), and butyryl-CoA dehydrogenase (*bcd*) [57].

Our analysis revealed that multiple butyrate-related genes were depleted in the GM of patients with MASLD (Fig. 2B). In Cohort 2, the genes *bcd*,* but* and *fabV* showed reductions in abundance of roughly 1.5- to 5-fold in advanced MASLD compared with early-stage disease, while *ptb* and *buk* showed no significant differences. Similarly, in Cohort 3, *bcd*, *but*, *ptb*, and *buk* were 1.5- to 2-fold less abundant in cirrhosis compared to healthy controls. Among these, *fabV* was nearly tenfold less abundant than *bcd* and *ptb*, indicating a minor contribution of this pathway in butyryl-CoA synthesis relative to the dehydrogenase route. Notably, no depletion of butyrate genes was observed in Cohort 1, suggesting that functional disruption becomes more evident with disease progression.

### Microbial ethanol and propanol pathways are increased in MASLD

Given the known metabolic and inflammatory effects of microbial ethanol and propanol, we hypothesized that genes encoding short-chain alcohol (SCA)- producing enzymes may represent an additional functional signature associated with MASLD progression. These metabolites are formed by the GM via fermentative reduction of their corresponding aldehydes through reactions that not only detoxify reactive intermediates but also regenerate NAD^+^, sustaining bacterial growth under anaerobic conditions [58, 59].

**Fig. 3:**
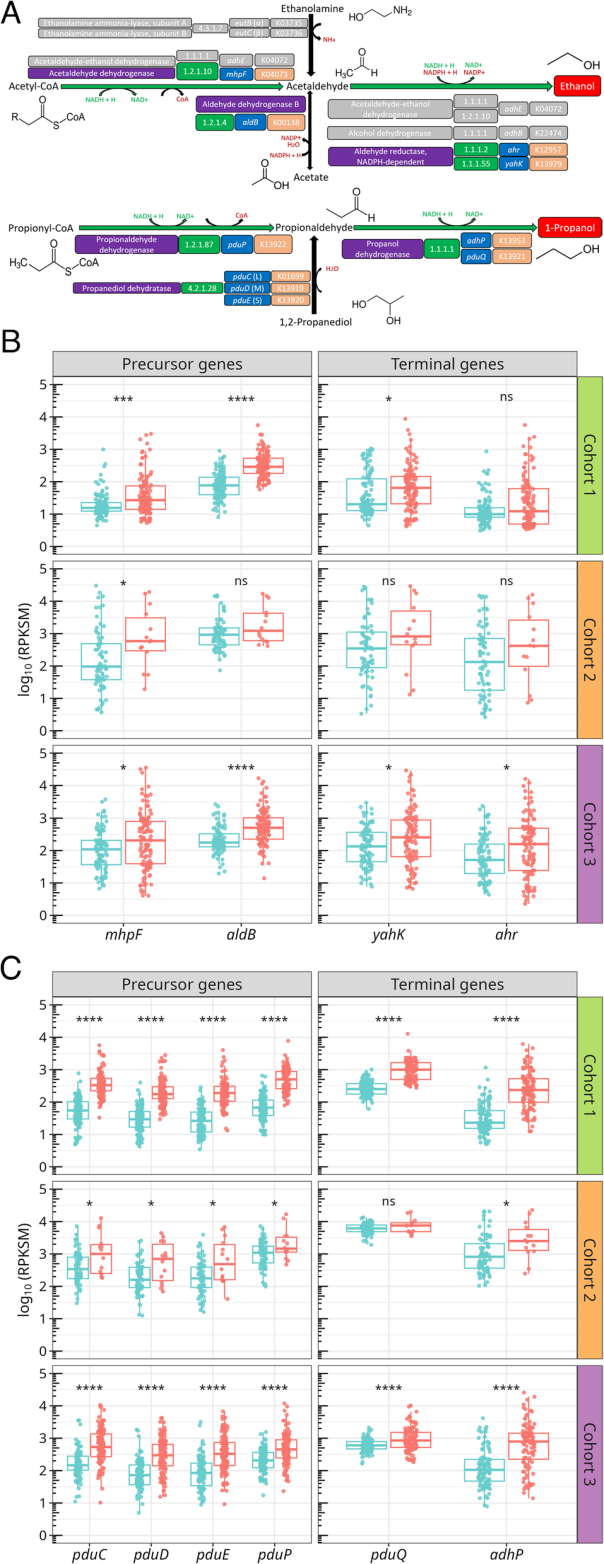
Abundance of SCA-producing genes in MASLD. **A** Terminal reactions involved in the formation of bacterial ethanol (top) and propanol (bottom). Enzymes, EC numbers, coding genes and KOs, as well as reaction types, are indicated according to the legend in Fig. 2A. Non-candidate genes are colored in grey. **B** Abundance of *mhpF*,* aldB*,* yahK* and *ahr* genes involved in the final reactions from pyruvate to ethanol across the three cohorts. **C** Abundance of *pduC-E*,* pduP*,* pduQ* and *adhP* genes, involved in the last reactions from 1,2-propanediol and propionyl-CoA to 1-propanol. Boxplot layout and facet distribution, as well as statistical tests, were performed as in Fig. 2B

Bacterial ethanol is formed from acetaldehyde through a fermentative reaction that can be catalyzed by (1) an NADH-dependent, bifunctional acetaldehyde-ethanol dehydrogenase (*adhE*), (2) an NADPH-dependent alcohol dehydrogenase (*adhB*) [60] and (3) a NADPH-dependent aldehyde reductase encoded by two different genes: *ahr* and *yahK*, which regenerate NADP^+^ [61, 62]. Acetaldehyde can be generated from (1) ethanolamine degradation, via ethanolamine ammonia-lyase (*eutB* and *eutC*) [63], (2) acetyl-CoA, through bifunctional AdhE or CoA-acetylating acetaldehyde dehydrogenase (*mhpF*) [64, 65] and (3) acetate, via aldehyde dehydrogenase B (*aldB*) [66] (Fig. 3A, top).

Bacterial 1-propanol is formed from propionaldehyde through a propanol dehydrogenase encoded by *adhP* and *pduQ.* Propionaldehyde can be generated by (1) a CoA transfer from propionyl-CoA catalyzed by a propionaldehyde dehydrogenase (*pduP*) and (2) a dehydration of 1,2 propanediol via propanediol dehydratase composed by three subunits (*pduC*, *pduD* and *pduE*). These genes are co-located in the same operon and form part of the propanediol-utilization (Pdu) microcompartment, a bacterial organelle found in certain enteric species [67] (Fig. 3A, bottom).

Our metagenomic profiling revealed that genes involved in ethanol (*yahK* and *ahr*) and acetaldehyde (*mhpF* and *aldB*) production were consistently enriched in MASLD patients compared with healthy controls (Fig. 3B). In Cohort 1, *mhpF* and *aldB* increased by approximately twofold, while *yahK* rose nearly fourfold; *ahr*, however, showed no significant differences. In Cohort 2, only *mhpF* displayed a marked elevation -approximately fivefold higher in advanced versus early MASLD- whereas the other genes showed non-significant increases along the fibrosis continuum. In Cohort 3, all four genes (*mhpF*, *aldB*, *yahK*, and *ahr*) were enriched in cirrhosis, typically by one- to threefold relative to healthy individuals. Among them, *mhpF* and *ahr* were consistently less abundant than their counterparts *aldB* and *yahK*, respectively. Additionally, all propanol-related genes examined (*pduCDE*, *pduP*, *pduQ* and *adhP*) were significantly more abundant in MASLD patients compared to healthy controls (Fig. 3C, Supplementary Fig. 4 A). In Cohort 1, these genes showed increases ranging from roughly sixfold (*pduCDE*) to over tenfold (*pduP*,* pduQ* and *adhP*). In Cohort 2, their abundances rose progressively with fibrosis severity, with *pduCDE* and *pduP* increasing three-to sixfold, and *adhP* showing the strongest elevation -up to fourfold higher in advanced vs. early MASLD- while *pduQ* remained stable. In Cohort 3, all genes were again enriched in cirrhosis, typically by two- to fivefold relative to healthy individuals. Together, these findings reveal a systematic and stage-associated enrichment of genes involved in the microbial production of ethanol and propanol in MASLD.

### MASLD is marked by decreased methanogenesis and increased TMA regeneration

Trimethylamine N-oxide (TMAO) is a hepatic oxide product of trimethylamine (TMA), a microbial metabolite derived from dietary choline and L-carnitine. Circulating TMAO levels have also been linked to MASLD severity [68] and all-cause mortality in MASLD patients [69]. Therefore, we investigated whether microbial genes involved in TMA and TMAO metabolism might reveal additional functional disruptions contributing to MASLD pathophysiology.

**Fig. 4:**
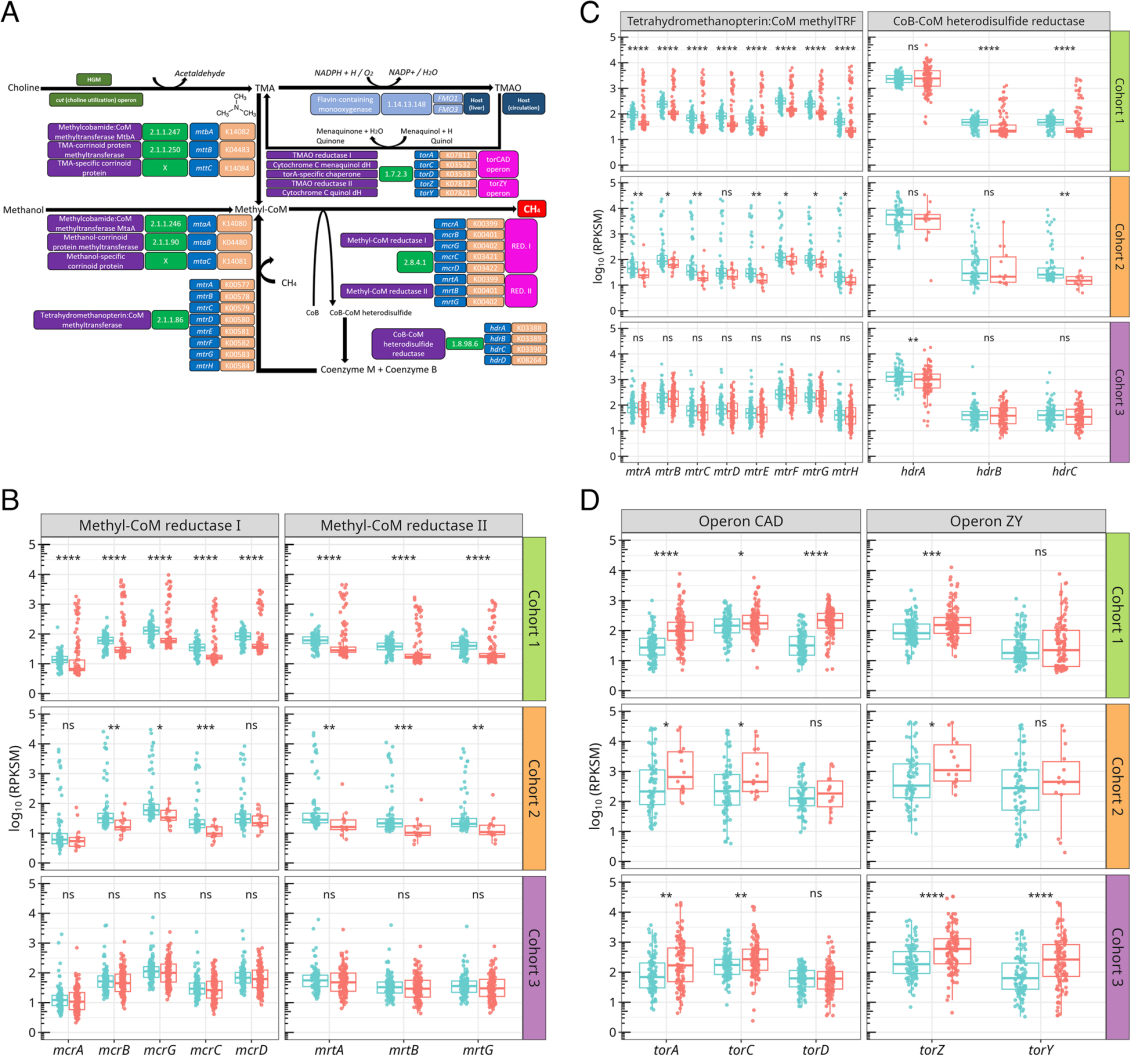
Abundance of methane and TMA-producing genes in MASLD.** A** Terminal reactions involved in the formation of microbial methane. Enzymes, EC numbers, coding genes and KOs, as well as reaction types, are indicated according to the legend in Fig. 2A. **B** Abundance of methyl-CoM reductase genes (*mcr*, MCR I; *mrt*, MCR II) across cohorts. **C** Abundance of *hdr* and *mtr* operons, involved in cofactor regeneration and methyl-CoM replenishment, respectively. **D** Abundance of *tor* operons (*torCAD* and *torZY*), responsible for TMA regeneration from TMAO. Facets group genes by operon. Boxplot layout and facet distribution, as well as statistical tests, were performed as in Fig. 2B

TMA is generated in the GM from choline catabolism via the *cut* operon [70] and can follow two divergent fates. Firstly, TMA can be oxidized in the liver to TMAO [71] which, under anaerobic conditions, can be further reduced back to TMA via the *torCAD* and *torZY* operons, which encode two TMAO reductases, *torA* and *torZ*, with distinct catalytic mechanisms [72] (Fig. 4A). Secondly, in methanogenic microbes, TMA can also serve as a substrate for methane production via sequential methylation of coenzyme M (CoM) and subsequent reduction steps. Methanogenesis from TMA involves methyl-CoM formation through TMA- and methanol-specific pathways (*mtt* and *mta* operons) [73], followed by its reduction to methane via methyl-CoM reductases (MCRs) encoded by *mcr* (MCR I) and *mrt* (MCR II) operons [74, 75]. The resulting heterodisulfide (CoB-S-S-CoM) is recycled back to its cofactors by a heterodisulfide reductase (*hdr* operon) [76], while methyl-CoM is replenished by tetrahydromethanopterin CoM-methyltransferase (*mtr* operon) [77].

Metagenomic analysis revealed that genes encoding MCR I and II were significantly decreased in MASLD patients and declined with disease progression within Cohort 2 (Fig. 4B, Supplementary Fig. 4B). In Cohort 1, median abundances were roughly 2–4-fold lower in MASLD relative to healthy controls. In Cohort 2, both MCR I and II genes showed more modest reductions, typically around 1–2-fold between early- and advanced-stage MASLD. No significant differences were detected in Cohort 3. Concordantly, *hdr* and *mtr* genes were depleted in MASLD patients relative to healthy controls, showing reductions with analogous magnitudes in Cohorts 1 and Cohort 2, respectively (Fig. 4C). TMA- and methanol-specific genes (*mtbA*,* mttB*,* mtaA* and *mtaB*) also showed lower abundance, though without clear stage-dependent differences (Supplementary Fig. 5). Nonetheless, the abundance of methanogenic taxa was barely detected across all cohorts (Supplementary Fig. 6). Conversely, *torCAD* and *torZY* operons involved in TMA regeneration were significantly enriched in MASLD and increased with fibrosis severity between 1–3-fold (Fig. 4D). This suggests a metabolic rerouting in the GM favoring TMA regeneration over methane synthesis, which may contribute to increased hepatic TMAO load and pro-inflammatory signaling in MASLD.

To rule out technical or biological confounders, we quantified five universal single-copy genes (USCGs) as controls. No significant differences in USCG abundance were detected across groups in any cohort (Supplementary Fig. 7).

### Metabolic gene distribution reveals functional plasticity in the GM

**Fig. 5:**
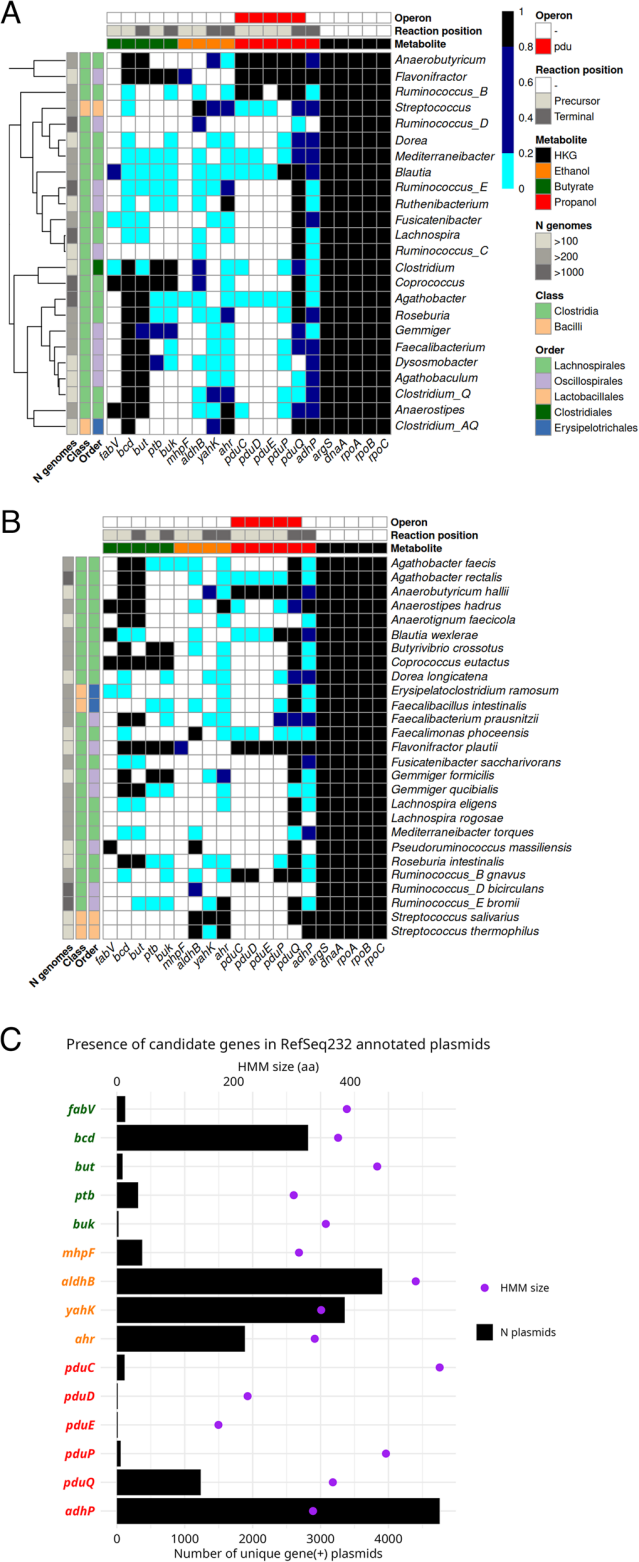
Presence of candidate metabolic genes in the human GM.** A-B** Heatmaps showing the presence of MASLD-associated genes across some of the most abundant clades in the human GM. The x-axis represents genes associated with butyrate and SCA production, as well as USCGs. The y-axis lists the bacterial clades inspected. Gene presence is expressed as the percentage of (**A**) congeneric and (**B**) conspecific genomes encoding each gene. Colors indicate prevalence in both heatmaps: black (core, > 80%), dark blue (accessory, 20–80%), light blue (highly accessory, < 20%) and white (absent, 0%). Genera are hierarchically clustered on the y-axis by gene-presence similarity. **C** Distribution of candidate genes across RefSeq232 plasmids. Bars represent the number of distinct plasmids encoding at least one copy of each gene

We evaluated the presence of candidate metabolic genes across some of the most abundant human GM genomes. Genes were classified as core if found in > 80% of genomes within a clade, accessory if found in 20–80%, and highly accessory if found in < 20%. Most selected GM genera (*n* = 24) belong to class Clostridia, specifically to orders Lachnospirales and Oscillospirales (Fig. 5A). Butyrate-producing genes from the butyryl-CoA pathway (*bcd* and *but*) were classified as core in nearly half of these genera, while those from the butyryl-P pathway (*ptb* and *buk*) were core only in *Flavonifractor*,* Clostridium* and *Coprococcus.* These genes were highly accessory or absent in genera such as *Lachnospira*,* Ruminococcus_B-E*,* Ruthenibacterium*,* Blautia*, *Fusicatenibacter*, *Dorea*, and *Mediterraneibacter*.

Acetaldehyde- and ethanol-producing genes were highly accessory except in *Streptococcus*, a heterolactic genus in which *aldhB* was constitutively present (specifically in *S. salivarius* and *S. thermophilus*, Fig. 5B) and *yahK*/*ahr* were moderately present (20–80%). The acetaldehyde-producing gene *mhpF* was nearly absent in the GM, detected only in a few genomes of *Flavonifractor* and *Agathobacter*. Propionaldehyde- and propanol-producing genes from the *pdu* operon were core in *Ruminococcus_B* (*R_B. gnavus*), *Anaerobutyricum* (*A. hallii*) and *Flavonifractor* (*F. plautii*), although *pduE* was unexpectedly absent in *Ruminococcus_B*. These genes were highly accessory in *Blautia* (*B. wexlerae*), *Streptococcus*, *Mediterraneibacter* and *Agathobacter* (*A. rectalis*). Notably, *A. hallii* and *F. plautii* were the only species encoding core butyrate- and propanol-producing genes.

We also assessed the presence of candidate metabolic genes in plasmids to evaluate their potential for horizontal gene transfer (Fig. 5C). Ethanol-related genes (*aldhB*,* yahK*,* ahr*) were broadly distributed across plasmids, suggesting high mobility. In contrast, butyrate- and propanol- producing genes were found in far fewer plasmids, supporting the idea that these pathways are more genome-specific and chromosomally encoded. An exception was *bcd*, which was broadly present across both plasmids and chromosomes. These findings support the idea that non-essential metabolic functions, especially ethanol production, are often encoded in plasmids.

## Discussion

MASLD is a multifactorial liver pathology where the GM is increasingly recognized as a key modulator. However, efforts to define consistent taxonomic signatures have been hindered by conflicting findings across cohorts and the limited strain-level resolution of 16S rRNA profiling [78]. Our study addresses these limitations by applying gene-centric metagenomic profiling across three different cohorts with MASLD patients and healthy subjects, revealing a consistent and functional reprogramming of the GM in MASLD patients that is decoupled from taxonomic composition. We show that functional pathways associated with butyrate and methane production generally exhibit lower abundance across cohorts, though the magnitude of these differences varies. In contrast, microbial pathways for SCA and TMA production are more consistently enriched. These findings underscore the need to move beyond taxonomy and adopt function-centric approaches to elucidate microbiome contributions to liver disease.

While prior studies have reported taxonomic associations with MASLD, results have often been inconsistent across populations. For instance, *Ruminococcus* and *Prevotella* have been described as both increased and decreased depending on disease stage or cohort [14–16, 19]. Other genera, such as *Blautia* or *Roseburia*, have also shown discordant taxonomic signatures in MASLD [17, 20]. We improved taxonomic resolution by profiling multiple marker genes using MetaPhlan on fecal metagenomic data from healthy and MASLD donors across three independent cohorts. *Agathobacter rectalis* emerged in our analysis as a robust taxonomic marker, significantly depleted in MASLD patients across all cohorts. Although previously associated to MASLD [14, 19], the taxonomic ambiguity of *A. rectalis* (formerly *Eubacterium rectale* [79]) complicates comparisons across studies [80]. Given its butyrate-producing capacity, the consistent reduction of *A. rectalis* may signal a broader loss of beneficial microbial functions.

Given the role of GM metabolism in disrupting GLA integrity, we isolated and quantified the abundance of GM gene families responsible for producing metabolites previously associated to MASLD: butyrate, SCAs, TMA and methane [81–83]. Butyrate-producing genes, particularly those of the crotonyl-butyryl CoA axis (i.e., *fabV*,* bcd*,* but*) were depleted in MASLD. These differences were more pronounced in cirrhosis. Genes of the butyryl-P pathway (*ptb*,* buk*) showed milder, non-significant differences across MASLD stages. These findings are consistent with the notion that the CoA transfer pathway is the predominant route for butyrate production in the GM [84]. Butyrate is known to preserve gut barrier integrity and exert anti-inflammatory effects in the liver, and its depletion likely contributes to GLA disruption and increased susceptibility to MASH [85–87].

Contrarily, ethanol- and propanol- producing genes, including aldehyde dehydrogenases (*mhpF*,* aldB*), reductases (*yahK*,* ahr*), *adhP* and the *pdu* operon (*pduCDE*,* pduP* and *pduQ*), were significantly increased in MASLD. These results suggest a metabolic shift towards fermentative alcohol production, possibly driven by an increased abundance or activity of facultative anaerobes. Previous clinical studies have reported elevated blood ethanol and increased fecal *Escherichia* 16S rRNA levels in MASH patients [88–90], and high-alcohol-producing strains of *Klebsiella pneumoniae* have been causally linked to MASLD in murine models [91, 92]. Importantly, while endogenous ethanol production has been linked to MASLD pathogenesis in both human and murine studies, our findings suggest that 1-propanol may also play a role. Genes for propanol biosynthesis, particularly those produced in bacterial microcompartments such as the *pdu* operon, were highly enriched and may play a more significant role than ethanol in MASLD. The structural and metabolic parallels between ethanol and propanol pathways warrant further investigation, especially given their potential to disrupt hepatic lipid metabolism and oxidative homeostasis.

We also identified an imbalance in microbial TMA and methane metabolism. Methanogenesis genes -including *mcr* (MCR I), *mrt* (MCR II), *mtr*,* hdr*- were overall depleted in MASLD. However, a subset of early-stage MASLD individuals exhibited markedly higher abundances of these genes, suggesting potential bimodality within this group. This variability may reflect distinct microbial configurations or disease etiologies, pointing to the existence of MASLD subgroups with divergent gut metabolic profiles. In contrast, TMAO-reductive genes *(torCAD* and *torZY*), which convert TMAO to TMA under anaerobic conditions, were enriched in MASLD. This pattern suggests a metabolic bottleneck that impairs TMA conversion to methane and instead favors recycling of TMA, potentially increasing hepatic exposure to TMAO. Given that TMAO has been linked to insulin resistance, inflammation and cardiovascular risk [93, 94], these results underscore how interindividual variability in gut methanogenesis may influence the metabolic trajectory of MASLD and highlight the need for precision approaches in its management.

Collectively, our results reveal a geno-metabolic shift in MASLD, characterized by an increased capacity for SCA and TMA production and decreased potential for butyrate and methane synthesis. These differences were independent of overall microbial load, and support the hypothesis that the accumulation of endogenous alcohols is a key metabolic feature of MASLD, occurring alongside the displacement of butyrate-producing taxa [95]. Notably, this pattern was partly reproduced in the whole-metagenome analysis performed on random sub-cohorts, particularly for genes involved in the TMA-methane metabolic axis and propanol production, supporting the robustness of our gene-centric results. Minor discrepancies likely reflect the reduced sample size of the co-assemblies and the broader KO definitions used in that complementary approach.

Crucially, many of the genes identified as altered in MASLD were either highly accessory or plasmid-encoded. Ethanol-related genes (*aldB*, *yahK*, *ahr*) exhibited broad plasmid distribution, suggesting that horizontal gene transfer plays a role in disseminating fermentative capabilities across gut taxa. In contrast, butyrate and propanol genes were more often chromosomally encoded and restricted to a narrower phylogenetic range. This differential mobility is in line with the idea that non-essential, adaptive functions are often carried on plasmids [22, 96–98]. As a result, functionally distinct genomic variants may arise within the same species [32], which often harbor different metabolic genes such as those involved in fermentative pathways from lactobacilli or enterobacteria [99, 100]. This underscores the limitations of taxonomy alone in disease associations, as genome plasticity leads to divergent metabolic capacities within species, a well-documented effect in both pathogenic and commensal *E. coli* strains [98, 101].

Our findings underscore the importance of microbial metabolism, rather than taxonomy, as a driver of MASLD-related gut-liver crosstalk. The identification of consistently altered metabolic pathways offers mechanistic insight and opens new avenues for interventions. For instance, the loss of butyrate-producing capacity could be addressed through targeted probiotic or dietary therapies, while strategies to reduce microbial alcohol or TMA production (e.g. bacteriophage targeting or metabolic inhibition) may limit hepatic damage. The strain-level resolution enabled by genome-resolved metagenomics also sets the stage for microbiome-based precision medicine approaches in MASLD.

Our study has several limitations. First, although gene abundance is a proxy for functional potential, it does not directly measure metabolic activity. Integration with metatranscriptomics or metabolomics would provide complementary insight into gene expression and metabolite production. Second, while our study provides strong cross-cohort evidence of functional shifts in MASLD, it does not establish causality. Experimental validation, including microbial isolation, germ-free transfer models, or gene knockouts, will be needed to clarify mechanistic roles. Moreover, the role of diet, medications, and host genetic background warrants further exploration, as these factors may modulate the availability and activity of the implicated microbial genes.

## Conclusions

Our study reveals that MASLD is characterized by distinct geno-metabolic shifts in the GM that favors the production of endogenous alcohols and TMA, while depleting protective pathways for butyrate and methane synthesis. These alterations are largely driven by strain-level variation and MGEs, underscoring the limitations of taxonomy-based microbiome profiling. Gene-centric and mobile element-aware approaches reveal functional microbial signatures of MASLD progression and offer new targets for microbiome-based diagnostics and therapeutics.

## Supplementary Information

Supplementary Material 1.

## Data Availability

The metagenomic datasets analyzed during the current study are available in the Sequence Read Archive repository, with the following accession numbers: PRJNA544527, PRJEB14215, PRJNA420817, PRJNA373901 and PRJEB6337. Associated code is available in: https://github.com/JuanmaMedina/MASLD_FG_paper. Any additional information required to reanalyze the data reported in this paper is available from the lead contact upon request.

## Abbreviations

EC: Enzyme commission
GLA: Gut-liver axis
GM: Gut microbiome
KO: KEGG Orthology
MASLD: Metabolic dysfunction-associated steatotic liver disease
MASH: Metabolic dysfunction-associated steatohepatitis
MCR: Methyl-CoM reductase
MGE: Mobile genetic element
ORF: Open reading frame
Pdu: Propanediol-utilization
pHMM: Profile Hidden Markov Model
RPKSM: Reads per kilobase per family size per million reads
rRNA: Ribosomal rRNA
SRA: Sequence Read Archive
SCAs: Short-chain alcohols
SCFAs: Short-chain fatty acids
TMA: Trimethylamine
TMAO: Trimethylamine N-oxide
UHGG: Unified Human Gastrointestinal Genome
USCG: Universal single-copy gene
