# Supplementary material for: Bacterial metabolic signatures in MASLD predicted through gene-centric studies in stool metagenomes
Source: BMC Microbiol. 2025 Dec 18;26:70. doi: 10.1186/s12866-025-04549-5 (PMC12853991; doi:10.1186/s12866-025-04549-5)
Supplement: Supplementary file 1 — Supplementary Material 1. [file 12866_2025_4549_MOESM1_ESM.docx]

Supplementary material

**Bacterial metabolic signatures in MASLD predicted through gene-centric studies in stool metagenomes**

**
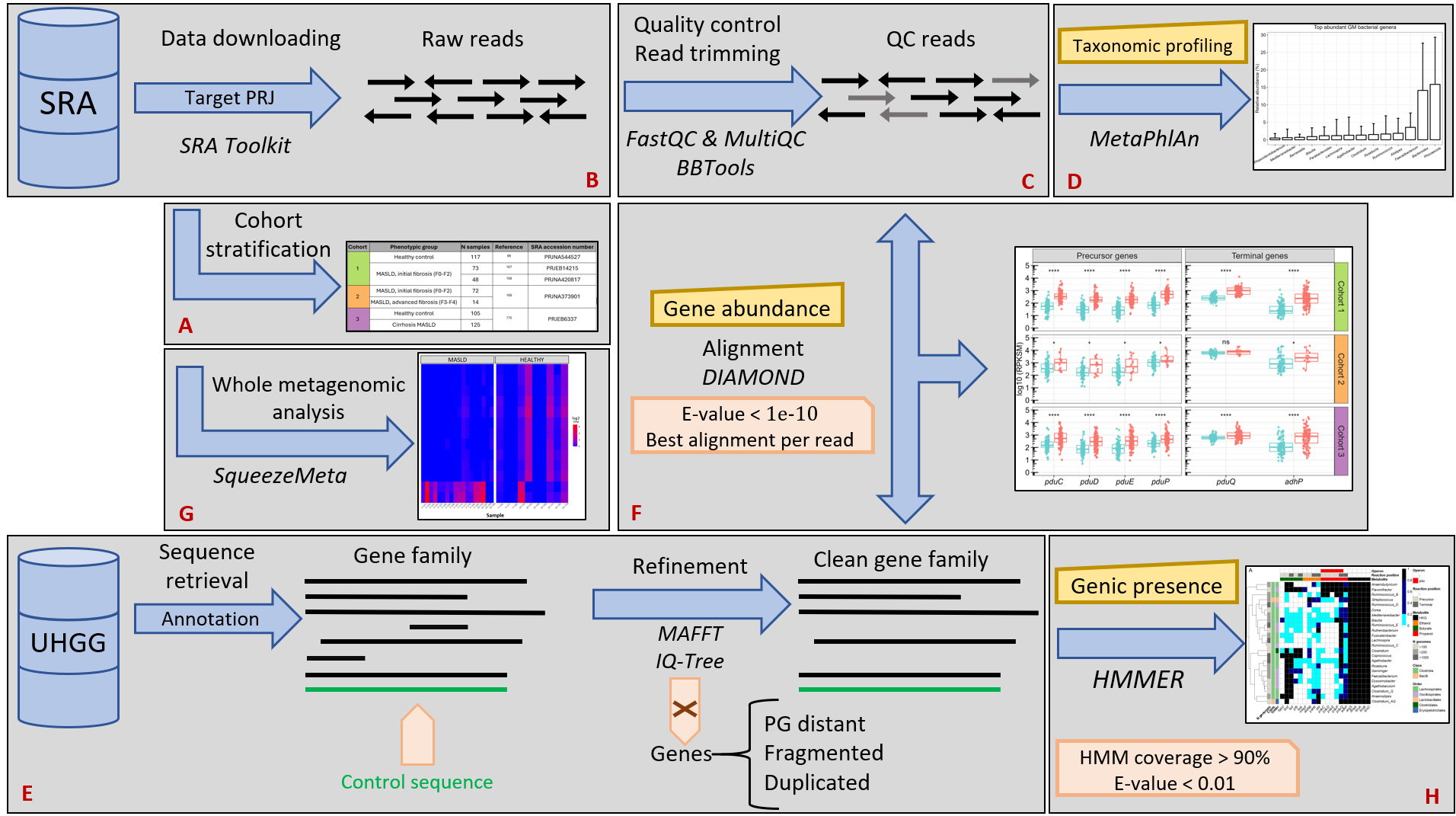
**

Supplementary Figure 1. Overview of the computational workflow used in this study. Diagram summarizing the full bioinformatic pipeline for processing paired-end metagenomic samples: from data retrieval and read quality control to taxonomic profiling and target gene quantification. Gene families were isolated and aligned against sequencing libraries to quantify abundance, and screened across GM genomes and annotated plasmids. Blue arrows indicate the workflow progression; software tools are shown in black below each arrow. Filtering steps are highlighted in pink boxes. Red labels refer to subsections (A-H) in Materials and Methods for detailed descriptions.

**
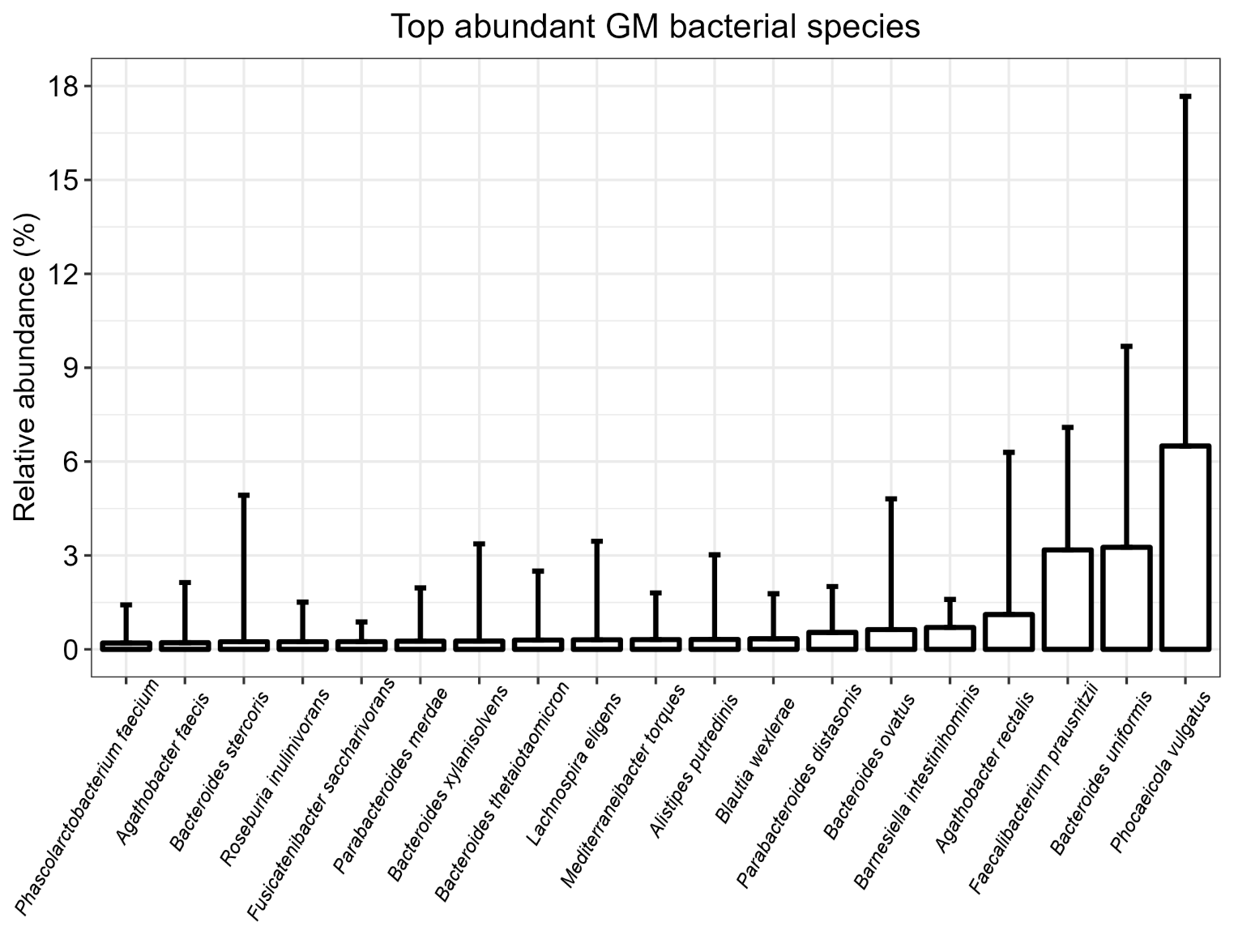
**

**Supplementary Figure 2. Top abundant GM species** (average absolute abundance >0.2%)

**
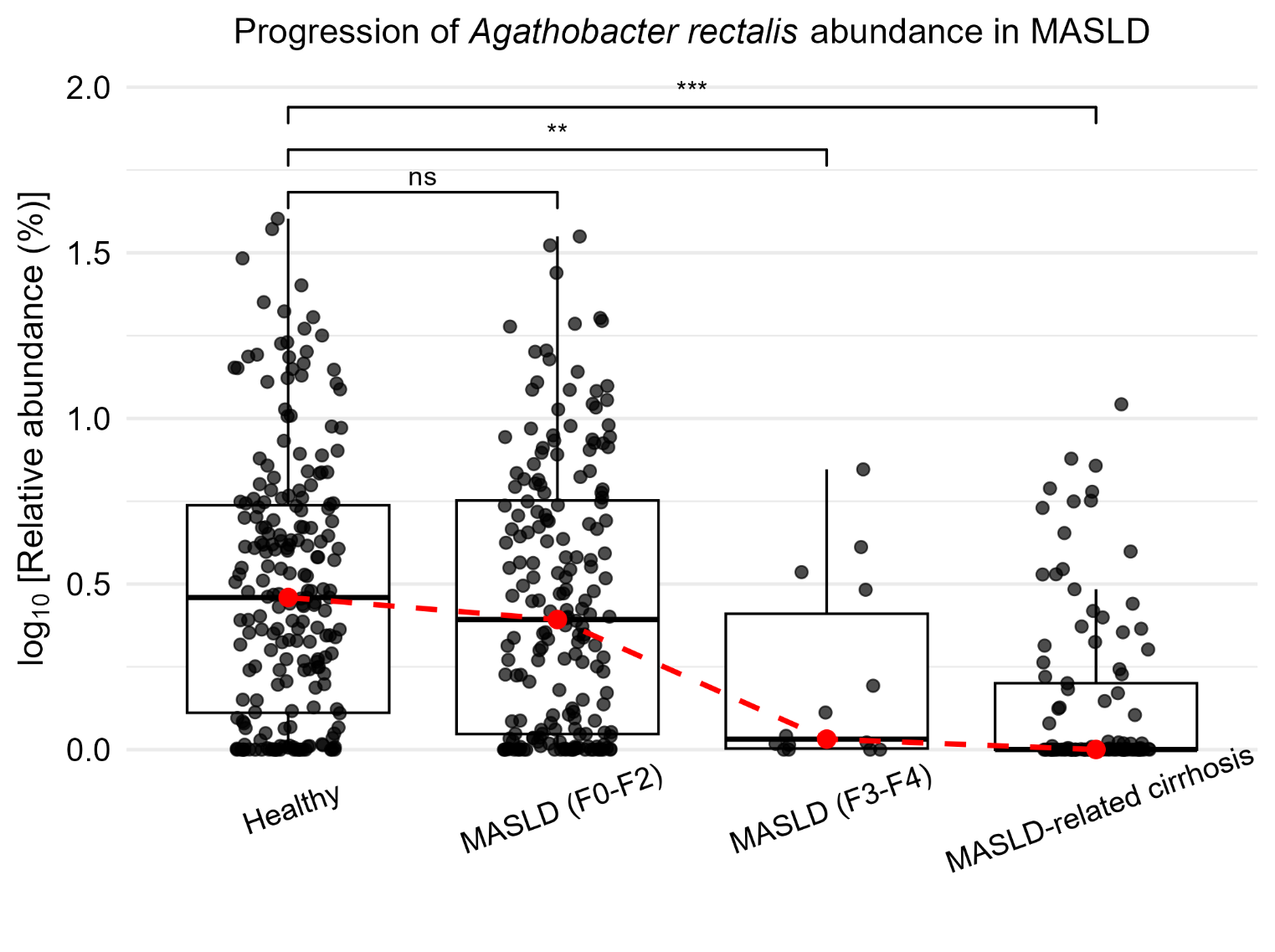
**

**Supplementary Figure 3. Abundance of *Agathobacter rectalis* in the GM across MASLD stages.** Abundances are shown on a logarithmic scale. Median values per group are connected by a red dashed line. Differences in abundance were evaluated using pairwise Mann-Whitney tests with Benjamini-Hochberg adjustment. Samples from all three cohorts were pooled, with individuals grouped into four clinical stages.


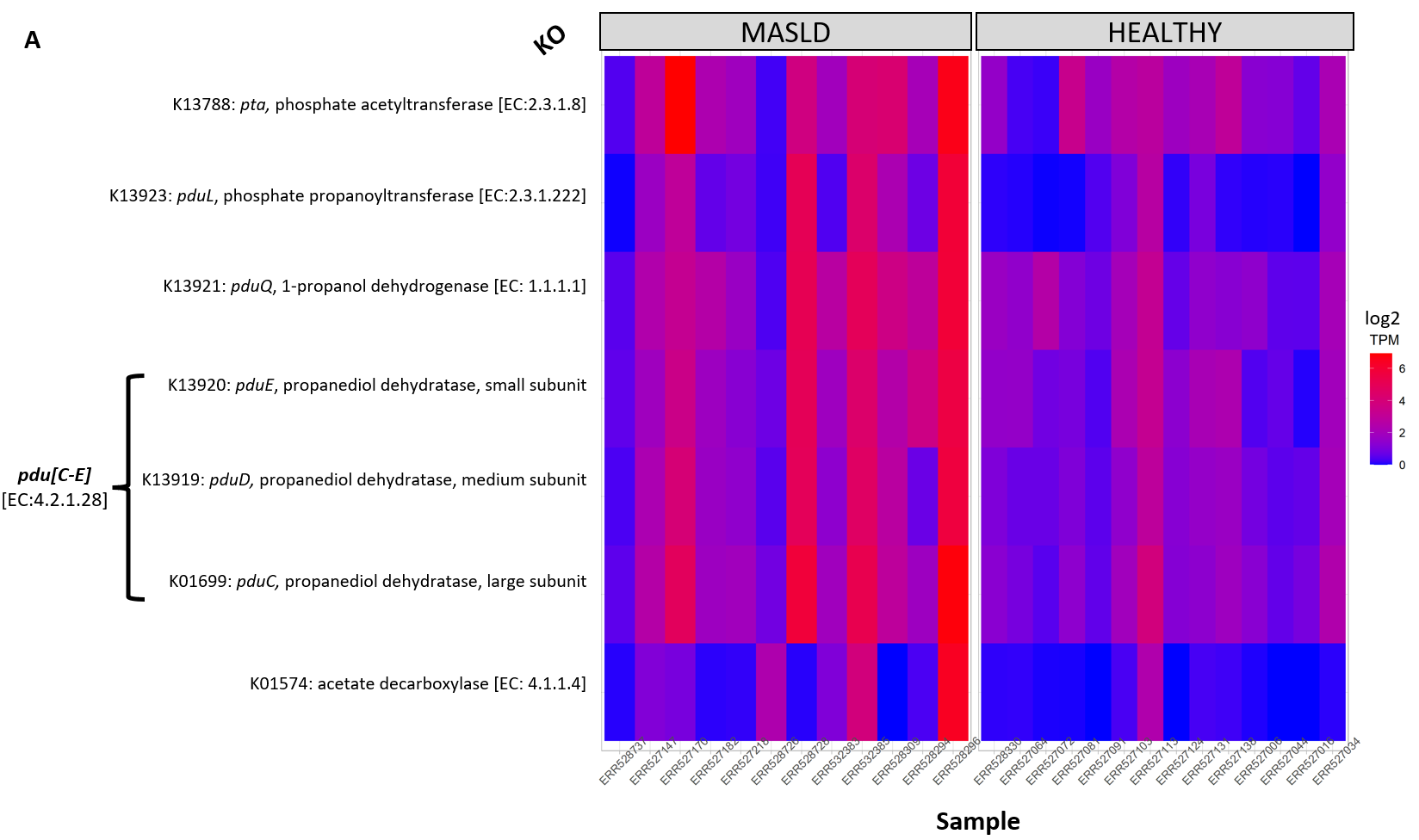

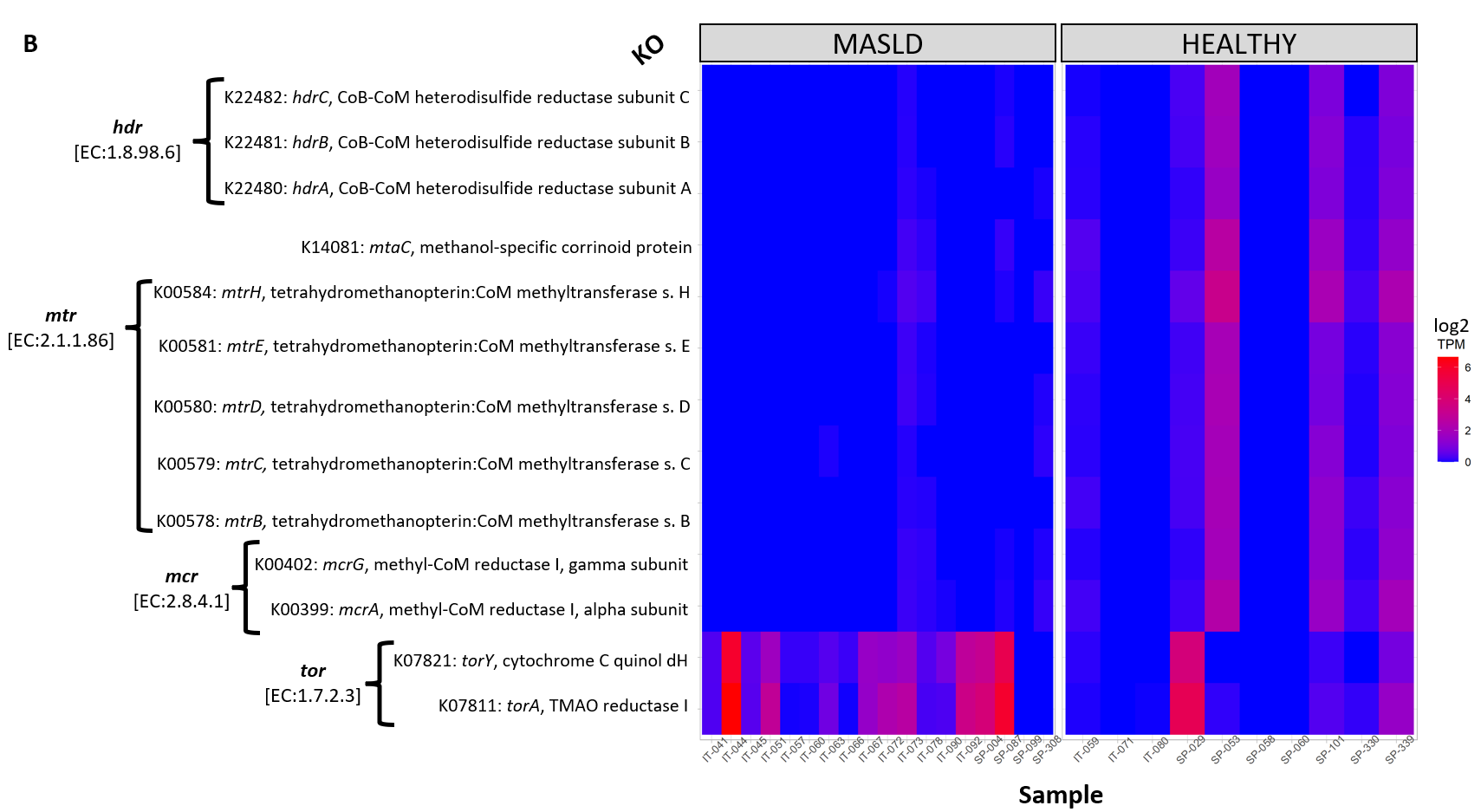


**Supplementary Figure 4. Abundance of candidate KO groups in MASLD.** Heatmaps represent KOs involved in the **(A)** propanol-formation and **(B)** TMA-methane metabolic pathway in sub-cohorts 3 and 1, respectively. KO abundance is expressed in tags per million (TPM), in log2 scale. x-axis indicates samples from both comparison groups and y-axis indicates KO groups, EC numbers, and associated gene and enzyme names according to KEGG. Only KOs with log fold-change >2 and a p-adjusted <0.05 are represented. KOs were predicted from the co-assembled sub-cohorts with SqueezeMeta.


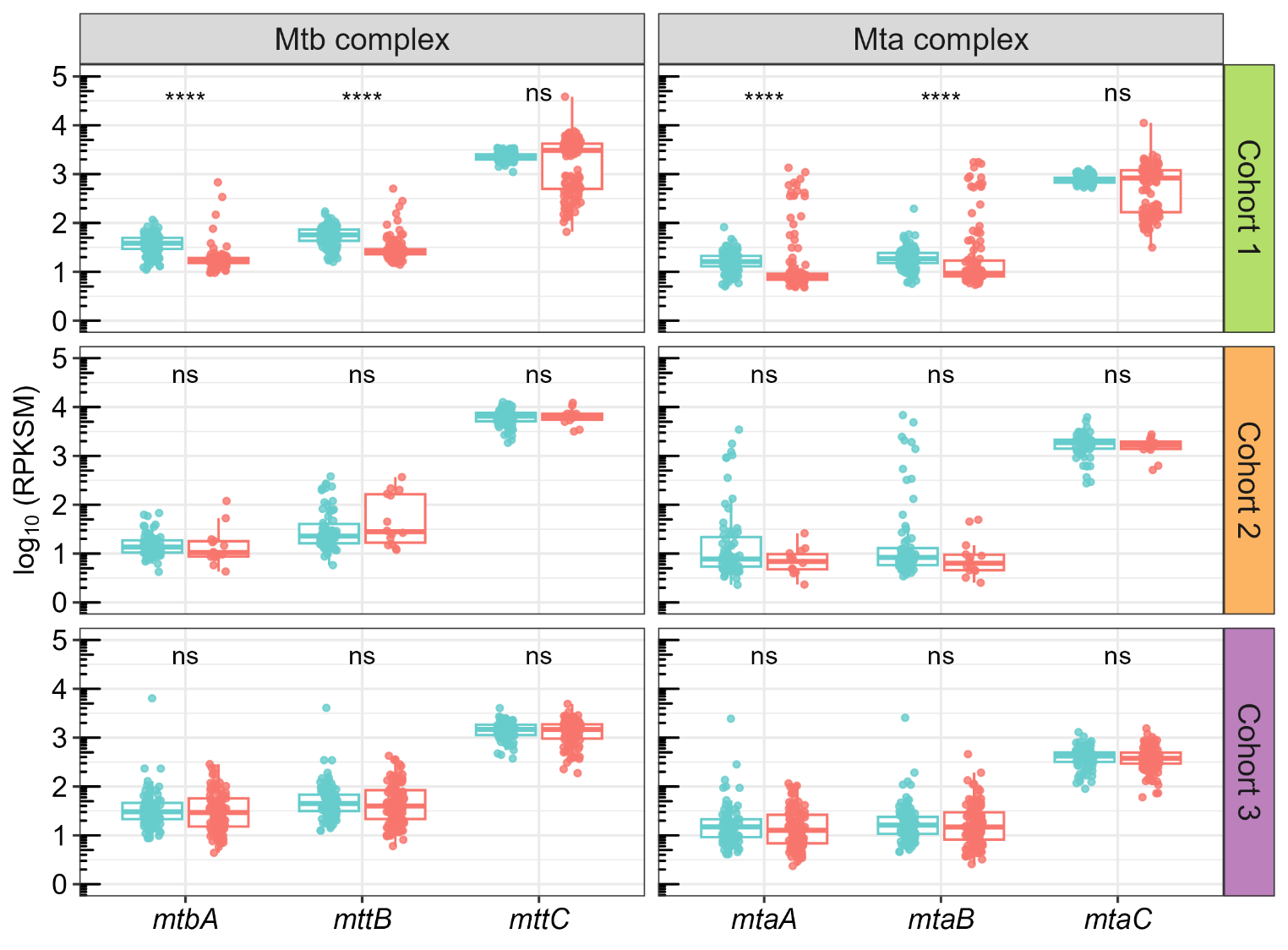


**Supplementary Figure 5. Abundance of genes coding for the Mtb and Mta complexes in patient cohorts.** Boxplot layout and facet distribution, as well as statistical tests, were performed as in Fig. 2B.


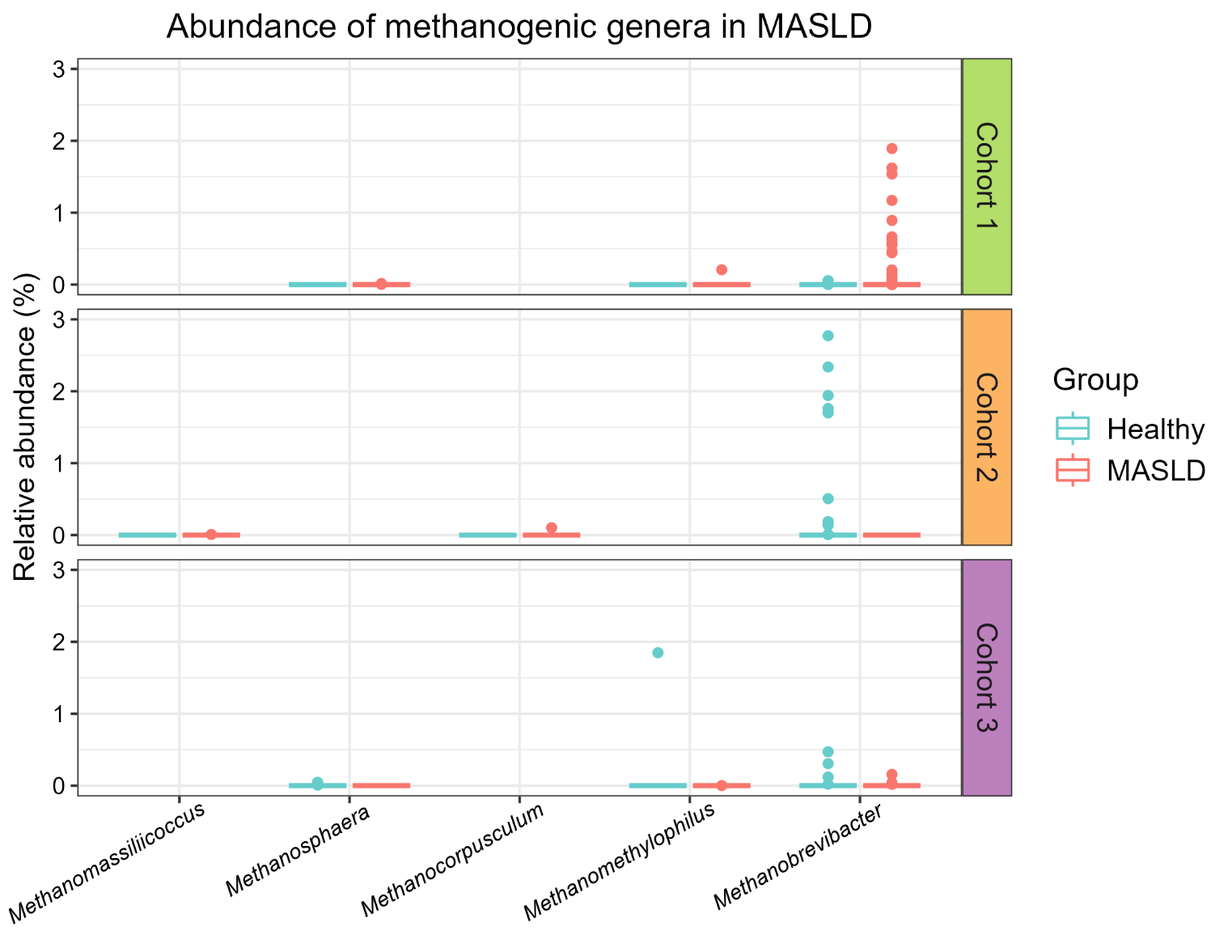


**Supplementary Figure 6. Abundance of methanogenic genera in MASLD.** Relative abundance of methanogenic clades in fecal metagenomes.


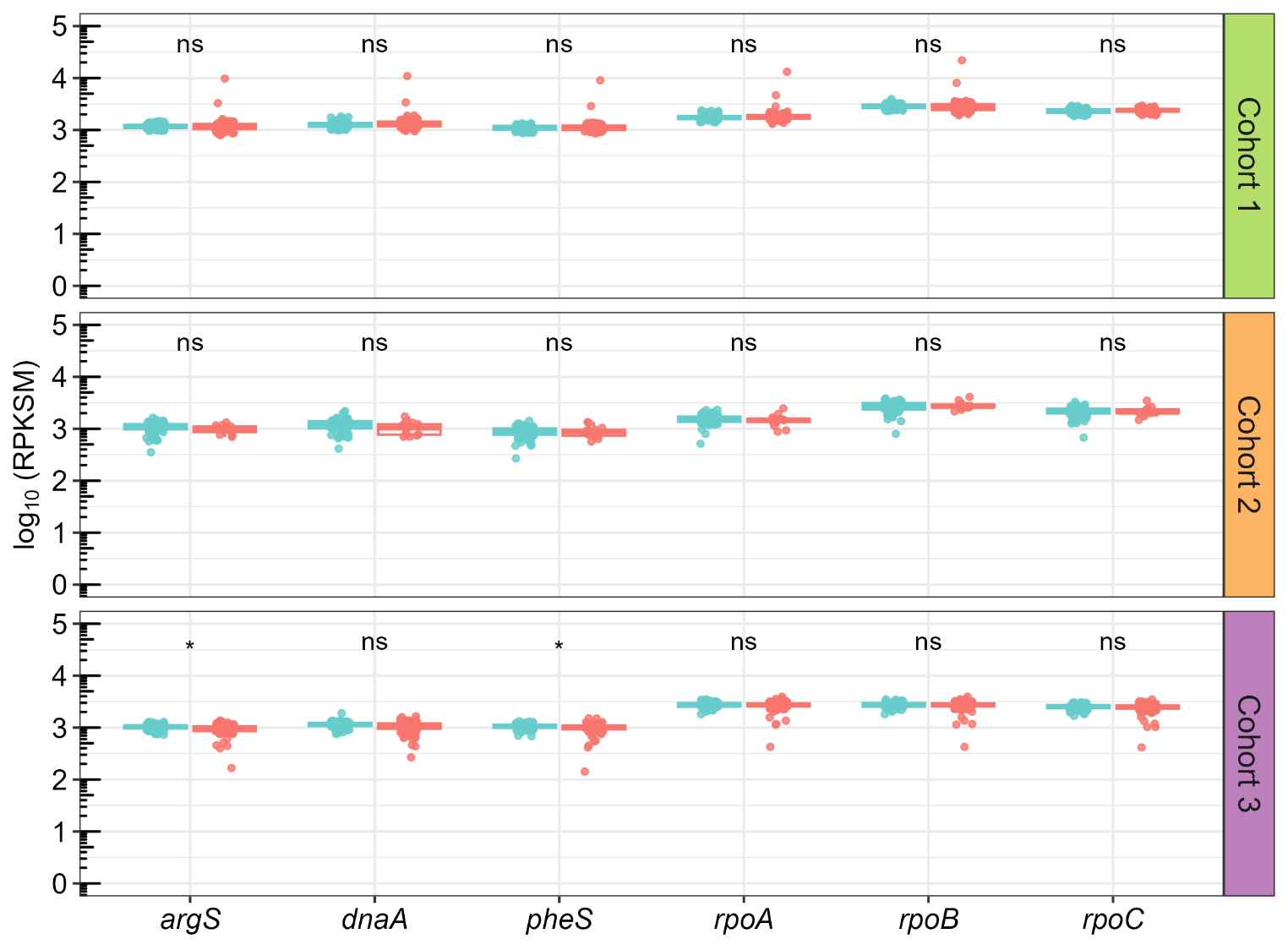


**Supplementary Figure 7. USCGs in patient cohorts.** Abundance of universal, single-copy marker genes in the three cohorts. Boxplot layout and facet distribution, as well as statistical tests, were performed as in Fig. 2B. *argS* encodes for the arginyl-tRNA synthetase*, dnaA* encodes for the chromosomal replication initiator protein DnaA and *rpoA/B/C* encode, respectively, the alpha, beta and gamma subunits of the DNA-directed RNA polymerase.
